# Post-operative recurrence of focal segmental glomerulosclerosis according to pre-transplant treatment after kidney transplantation

**DOI:** 10.1186/s12882-023-03098-1

**Published:** 2023-03-15

**Authors:** Hye Eun Kwon, Young Hoon Kim, Sang Ah Lee, Jae Jun Lee, Youngmin Ko, Sung Shin, Joo Hee Jung, Frances S. Sung, Chung Hee Baek, Hyosang Kim, Su-Kil Park, Hyunwook Kwon

**Affiliations:** 1 Division of Kidney and Pancreas Transplantation, Department of Surgery, Asan Medical Center, University of Ulsan College of Medicine, 88, Olympic-ro 43-gil, Songpa-gu, 05505 Seoul, Korea; 2 Division of Nephrology, Department of Internal Medicine, Asan Medical Center, University of Ulsan College of Medicine, Seoul, Korea

**Keywords:** Kidney transplantation, Focal segmental glomerulosclerosis, Plasmapheresis

## Abstract

**Background:**

Recurrent focal segmental glomerulosclerosis (FSGS) after kidney transplantation (KT) is a serious complication and a significant risk factor for graft failure. However, there is no clear evidence of the effectiveness of pre-transplant treatment using plasmapheresis (PP) or rituximab in preventing post-operative FSGS recurrence after KT.

**Methods:**

This single-center retrospective study included 99 adult patients with biopsy-proven primary FSGS who underwent KT between 2007 and 2018. The patients were divided into the pre-treatment group (N = 53, 53.5%) and no pre-treatment group (N = 46, 46.5%). In the pre-transplant group, prophylactic PP was administered before KT in patients undergoing living donor transplantation and the day after KT in those undergoing deceased donor transplantation.

**Results:**

The rate of immediate post-operative recurrence was significantly higher in the no pre-treatment group (16 [34.8%]) than in the pre-treatment group (5 [9.4%]; *P* = 0.002). There were three cases of graft failure due to recurrent FSGS, all of which were in the no pre-treatment group. After adjusting for possible confounding factors, age (per 10-year increase; OR = 0.61, CI, 0.42–0.90; *P* = 0.012) and pre-transplant treatment (vs. no pre-transplant treatment; OR = 0.17, CI, 0.05–0.54; *P* = 0.003) were identified as significant factors associated with FSGS recurrence. The rate of death-censored graft survival was significantly superior in the pretransplant treatment group (*P* = 0.042).

**Conclusion:**

Pre-transplant treatment with PP was associated with beneficial effects on preventing FSGS recurrence after KT.

## Introduction

Focal segmental glomerulosclerosis (FSGS) is one of the leading causes of end-stage renal disease. Recurrent FSGS after kidney transplantation (KT) is a serious complication and a significant risk factor for graft failure, resulting in an inferior graft survival rate compared with KT with other causes [1, 2]. The recurrence rates have a wide range (10–55%) in previous studies [1–7]. The known predictors of FSGS recurrence include age, race, donor type, disease’s rapid progression, and diffuse mesangial proliferation in kidney biopsy [7, 8]. However, it is still challenging to predict FSGS recurrence after KT because most of the previous studies had a small sample size to identify risk factors with sufficient statistical power and studies using large registries are limited by insufficient information [1–3].

Plasmapheresis (PP) is an effective therapeutic procedure for recurred FSGS and can achieve a remission rate of 57–90% [2, 6, 9]. The theoretical basis of applying PP as a treatment is the targeting of circulating permeability factors, [10] whose presence in recipients’ serum is associated with the alteration of glomerular permeability to albumin, resulting in the injury to the glomerular-filtration barrier and persistent proteinuria [6, 10]. Although a recent study suggested possible candidates of permeability factor (e.g., cardiotrophin-like cytokine 1, antiCD40 antibody, soluble urokinase-type plasminogen activator receptor), the exact pathophysiologic mechanism of FSGS is still unclear [11]. Rituximab (CD20 monoclonal antibody) seemed to induce podocyte stabilization, albeit with insufficient efficacy and safety [11]. Recent observational studies have reported conflicting results regarding the effects of pre-conditioning with PP or rituximab [3, 4, 6, 12]. In addition, the number of patients included in those studies was too small to have enough statistical power. Therefore, there is no consensus about pre-transplant treatment using PP or rituximab.

Our center performs more than 300 cases of KT per year, 20% of which are ABO-incompatible (ABOi) KT [13]. In this study, we evaluated the effectiveness of pre-transplant treatment in preventing post-operative FSGS recurrence after KT by comparing the results of patients with FSGS who were treated before transplant as a pre-conditioning for ABOi KT and those who received PP before KT due to the physician’s preference.

## Materials and methods

### Patients

This single-center retrospective study included patients over 18 years of age with biopsy-proven primary FSGS who underwent living or deceased donor KT at Asan Medical Center (Seoul, South Korea) between 2007 and 2018. Patients who fulfilled all of the following criteria were selected in this study: (1) presence of nephrotic-range proteinuria (> 3.0 g/day); (2) histological identification of FSGS; and (3) diffuse effacement of podocyte foot processes in a native kidney biopsy [5, 14, 15]. Patients with a family history, recurrence of FSGS after previous KT, and suspected secondary causes, such as severe obesity, long-standing diabetes, atrophic kidney, and infection, were excluded. However, genetic analyses to identify genetic FSGS was not performed in this study. Patients with secondary causes, such as server obesity, long-standing diabetes, atrophic kidney, and infection, were excluded. Patients were divided into the pre-treatment group and the no pre-treatment group. The pre-treatment group was defined as recipients who underwent PP with or without rituximab; the pre-transplant group consisted of 22 patients who underwent two sessions of prophylactic PP before living donor KT, 28 recipients with flow cytometry crossmatch (FCXM)-positive and ABOi KT who received desensitization using rituximab and PP, and 3 patients who underwent prophylactic PP the day after deceased donor KT. This study was performed after receiving approval from the institutional review board of our center (approval number: 2021 − 1561).

### Definition

The recurrence of FSGS was defined as reappearance of nephrotic-range proteinuria (> 3.0 g/day) in the absence of other causes after KT [14, 15]. Complete remission (CR), partial remission (PR), and no remission were defined as urine protein excretion < 0.3 g/day, 0.3–1.5 g/day, and > 1.5 g/day, respectively [16]. Immediate post-operative recurrence was defined as FSGS recurrence occurring within two weeks after transplantation. The primary endpoint of this study was the rate of post-transplant FSGS recurrent according to pre-transplant treatment.

### Desensitization and immunosuppression

Recipients in ABOi- and FCXM-positive KT received a single dose of rituximab (100–500 mg) two weeks prior to starting PP. PP was performed using a COBE Spectra (Gambro BCT, Lakewood, CO, USA). In ABOi KT, the target of PP was IgM Ab titers against blood groups A or B that were below 1:8 in accordance with the standard tube method in ABOi KT. For positive T-cell FCXM, PP was performed until T-cell FCXM became negative. In B-cell positive FCXM KT, the timing of transplant was determined based on the trend in DSA MFI values detected by the Luminex test because rituximab can cause a false-positive result in the XM test [17]. Either basiliximab or anti-thymocyte globulin (ATG) was administered for induction. ATG induction was used in immunologically high-risk patients but not in recipients treated with rituximab to prevent lethal infectious complications [12]. For maintenance, a calcineurin inhibitor, mycophenolic acid, and corticosteroid were administered to patients. Although tacrolimus was the primary calcineurin inhibitor, recipients older than 55 years who were treated with rituximab received cyclosporin [13]. The 22 patients who underwent prophylactic treatment due to the physician’s preference received two sessions of PP before KT. The three patients who underwent prophylactic PP following deceased donor KT received two sessions of PP within three days after transplantation.

### Statistics

All Statistical analyses were performed with SPSS version 18.0 (SPSS Inc., Chicago, IL, USA). Normally distributed continuous variables were evaluated by *t*-test and described as mean ± standard deviation (SD); non-normal variables were compared with Mann–Whitney *U* test and described as median (interquartile range [IQR]). Where appropriate, categorical data were compared by χ^2^ or Fisher’s exact test. Overall graft survival rates were analyzed using the Kaplan–Meier method and compared with the log-rank test. Univariate and multivariate logistic regression was performed to identify the predictors of FSGS following KT with conditional forward method. A stepwise multivariate regression test was also performed with variables that showed *P*-values < 0.1 in univariate logistic regression analysis to evaluate the effect of pre-transplant treatment on FSGS recurrence after transplantation. Odds ratios (ORs) and 95% confidence intervals (CIs) were estimated and *P*-values ≤ 0.05 were considered significant.

## Results

The baseline characteristics of the study patients are shown in Table 1. Of the 99 patients included in the analysis, 53 (53.5%) were in the pre-treatment group and 46 (46.5%) were in the no pre-treatment group. The no pre-treatment group had a significantly longer dialysis duration (64.5 vs. 18.6 months; *P* < 0.001) and a higher proportion of those who received deceased donor transplantation (56.5% vs. 5.7%; *P* < 0.001) than the pre-treatment group. More patients in the no pre-treatment group used tacrolimus rather than cyclosporin as a maintenance drug than the treatment group (87.0% vs. 64.2%; *P* = 0.011). Otherwise, there were no significant differences between the study groups.

**Table 1:** Baseline and clinical characteristics of the study patients

Characteristics	No pre-treatment (N = 46, 46.5%)	Pre-treatment (N = 53, 53.5%)	*P*-value
Mean age (years)	37.1 ± 16.2	38.0 ± 12.9	0.97
Female sex	22 (47.8)	19 (35.8)	0.23
Dialysis duration (months)	64.5 ± 55.0	18.6 ± 34.3	< 0.001
Time from FSGS to dialysis or transplantation (months)	72.3 ± 55.9	62.9 ± 68.1	0.73
Pre-emptive transplantation	5 (10.9)	12 (22.6)	0.12
Body mass index (kg/m^2^)	21.4 ± 4.0	22.7 ± 5.0	0.15
Pre-transplant treatment			0.001
Plasmapheresis only	–	24 (45.3)	
Plasmaphereses + rituximab	–	29 (54.7)	
ABO-incompatible	0 (0.0)	23 (43.4)	N/A
FCXM-positive	0 (0.0)	5 (9.4)	N/A
HLA-A, B, DR mismatch	3.0 ± 1.6	2.9 ± 1.7	0.88
PRA class I	8.8 ± 22.8	12.9 ± 26.0	0.41
PRA class II	10.5 ± 25.0	10.5 ± 23.6	0.99
Deceased donor	26 (56.5)	3 (5.7)	< 0.001
Living donor	20 (43.5)	50 (94.3)	
Calcineurin inhibitor			0.011
Tacrolimus	40 (87.0)	34 (64.2)	
Cyclosporin	6 (13.0)	19 (35.8)	
Induction			0.005
No	2 (4.7)	0 (0.0)	
Basiliximab	36 (83.7)	45 (100.0)	
Anti-thymocyte globulin	5 (11.6)	0 (0.0)	
FSGS recurrence*	16 (34.8)	5 (9.4)	0.002
Graft failure due to recurrent FSGS	3 (6.5)	0 (0.0)	0.10

Continuous data are presented as means ± standard deviations, and categorical data are presented as number (%)

Abbreviation: FSGS, focal segmental glomerulosclerosis; FCXM, flow cytometry crossmatch; PRA, panel reactive antibody

* FSGS recurrence: immediate post-operative recurrence

The rate of immediate post-operative recurrence was significantly higher in the no pre-treatment group (16 [34.8%] vs. 5 [9.4%]; *P* = 0.002). There were 3 cases of graft failure due to recurrent FSGS, all of which occurred in the no pre-treatment group. Eighteen patients received therapeutic PP only and three received PP with rituximab. The median time from KT to FSGS recurrence was 6 (4.5–18.0) days. The degree of maximal proteinuria after KT was significantly higher in patients who had recurred FSGS than in those without recurrence (7.2 vs. 2.7 g/day, *P* < 0.001). Among 21 cases of FSGS recurrence, CR, PR, and no remission were noted in 8 patients (39.1%), 10 patients (47.6%), and 3 patients (14.3%), respectively (Table 2).

**Table 2:** Treatment modalities and clinical courses for recurrent FSGS*

	No recurrence (N = 78, 78.8%)	Recurrence (N = 21, 21.2%)	*P*-value
Pre-transplant treatment			0.006
Plasmapheresis only	23 (29.5)	1 (4.8)	
Plasmaphereses + rituximab	25 (32.1)	4 (19.0)	
Plasmapheresis number	2.5 ± 0.9 (N = 46)	2.6 ± 0.9 (N = 5)	0.90
Post-transplant treatment			N/A
Plasmapheresis only	–	18 (85.7)	
Plasmaphereses + rituximab	–	3 (14.3)	
Time from transplantation to FSGS recurrence (days)	–	6 (4.5–18.0)	N/A
Post-transplant proteinuria (mg/day)			
Maximal proteinuria	2785.4 ± 1961.6	7247.7 ± 7133.8	< 0.001
Proteinuria at discharge	319.9 ± 228.4	479.6 ± 411.6	0.10
Treatment result			N/A
Complete remission		8 (38.1)	
Partial remission		10 (47.6)	
No remission		3 (14.3)	

Continuous data are presented as means ± standard deviations, and categorical data are presented as number (%)

Non-parametric data are presented as median (interquartile range)

Abbreviation: FSGS, focal segmental glomerulosclerosis; IQR, interquartile range* FSGS recurrence: immediate post-operative recurrence

**Fig. 1:**
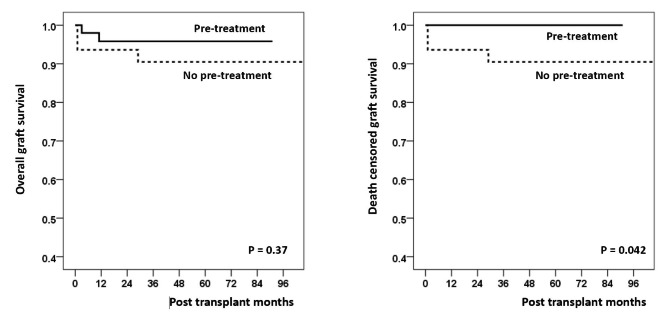
Long-term (A) overall survival rate and (B) graft survival rate

We performed univariate and multivariate logistic regression analyses to identify the risk factors for FSGS recurrence. In the univariate regression analysis, age (per 10-year increase; OR = 0.63, CI, 0.43–0.91; *P* = 0.014), deceased donor (vs. living donor; OR = 4.78, CI, 1.73–13.24; *P* = 0.003), and pretransplant treatment (vs. no pretransplant treatment; OR = 0.20, CI, 0.07–0.59; *P* = 0.004) were significantly associated with the risk of FSGS recurrence. After adjusting for possible confounding factors, age (per 10-year increase; OR = 0.61, CI, 0.42–0.90; *P* = 0.012) and pretransplant treatment (vs. no pretransplant treatment; OR = 0.17, CI, 0.05–0.54; *P* = 0.003) remained as significant factors associated with FSGS recurrence (Table 3). After stepwise introducing age (per 10-year increase), dialysis duration (per year), deceased donor (vs. living donor), and ABO incompatibility to multivariate analysis, pretransplant treatment (vs. no pretransplant treatment) showed an OR of 0.17(CI, 0.05–0.54; *P* = 0.003), 0.18 (CI, 0.05–0.61; *P* = 0.006), 0.24 (CI, 0.06–0.91; *P* = 0.036), and 0.25 (CI, 0.06–1.08; *P* = 0.063), respectively (Table 4). There was no significant difference between the pre-treatment group and the no pre-treatment group in long-term overall graft. However, the death-censored graft survival rate was significantly superior in the pre-transplant treatment group (*P* = 0.042). (Fig. 1)

**Table 3:** Risk factors associated with post-operative FSGS recurrence*

Variables	Univariate analysis		Multivariate analysis^$^		
	OR (95% CI)	*P*-value		OR (95% CI)	*P*-value
Age (per 10-year increase)	0.63 (0.43–0.91)	0.014	0.61 (0.42–0.90)		0.012
Female sex	0.76 (0.24–1.78)	0.40	N/A		N/A
Dialysis duration (per year)	1.10 (0.98–1.22)	0.10	N/A		N/A
FSGS to renal failure (per year)	0.98 (0.57–1.07)	0.57	N/A		N/A
Deceased (vs. living donor)	4.78 (1.73–13.24)	0.003	N/A		N/A
Pre-transplant treatment (vs. no treatment)	0.20 (0.07–0.59)	0.004	0.17 (0.05–0.54)		0.003
No pre-transplant treatment	Reference		N/A		N/A
Plasmapharesis only	0.08 (0.01–0.66)	0.019			
Plasmaphereses + rituximab	0.30 (0.09–1.01)	0.05			
Tacrolimus (vs. cyclosporin)	0.69 (0.22–2.17)	0.53	N/A		N/A
HLA-A, B, DR mismatch	1.13 (0.84–1.52)	0.42	N/A		N/A
PRA class I	1.01 (0.99–1.02)	0.66	N/A		N/A
PRA class II	1.00 (0.98–1.02)	0.92	N/A		N/A
ABO incompatibility	0.27 (0.06–1.25)	0.09	N/A		N/A

Abbreviation: FSGS, focal segmental glomerulosclerosis; PRA, panel reactive antibody

^$^Multivariate regression analysis was performed with the backward stepwise method

*FSGS recurrence: immediate post-operative recurrence

**Table 4:** Logistic regression models of risk factors associated with FSGS recurrence

Models	Pre-transplant treatment vs. no treatment	
	OR (95% CI)	*P*-value
Univariate model	0.20 (0.07–0.59)	0.004
Model 2 – univariate model + Age/10 years	0.17 (0.05–0.54)	0.003
Model 3 – Model 2 + Dialysis duration/year	0.18 (0.05–0.61)	0.006
Model 4 – Model 3 + cadaveric donor vs. living donor	0.24 (0.06–0.91)	0.036
Model 5 – Model 4 + ABO incompatibility	0.25 (0.06–1.08)	0.063

Abbreviation: FSGS, focal segmental glomerulosclerosis; CNI, Calcineurin inhibitor

## Discussion

In this study, we found that prophylactic PP was associated with a lower risk of immediate post-transplant FSGS recurrence. Among the traditional risk factors for recurrence FSGS, only young age significantly increased the recurrence rate in multivariate analysis. Rituximab alone did not show a significant preventable effect in our study. Among the 21 cases of post-operative recurrence, only 3 (14.3%) cases did not respond to treatment and ultimately experienced graft failure. Most recurrences in our study occurred within a month after transplant, suggesting that the early post-operative period was critical for improving graft survival in patients with FSGS. This finding was similar to the median time to recurrence of 1.25–1.5 months in the recent study [2, 12].

The recurrence and remission rates of our study are similar or lower than those reported in previous studies [2, 3, 5, 12, 18]. These outcomes must be interpreted with caution because the recipients in each study had varying baseline characteristics and underwent operations using different protocols. Most studies included in the recent meta-analysis and European registry data showed recurrence rates of 21–40%, which are similar to our study [18]. Alasfar et al. observed a much higher recurrence rate of 59%, [12] and also reported a tendency of efficacy but did not observe a significant difference in the post-operative recurrence rate according to preventative treatment. Such difference between our study and Alasfar et al. may be due to the fact that Alasfar et al. only included patients with high-risk idiopathic FSGS and the sample size was too small to reach enough statistical significance. A recent multicenter cohort study reported a 32% recurrence rate, [2] but the rate of treatment response in recurred patients was relatively lower than that in our study, with only 57% of patients achieving partial or complete remission [2]. The authors explained that the low remission rate likely resulted from nonstandardized treatment intensity and duration [2]. The relatively lower recurrence and higher remission rate in our study may have stemmed from the fact that more than half of the recipients had received prophylactic treatment. Furthermore, our center has implemented a protocol for FSGS in which recipients are not discharged until two weeks after KT while undergoing daily exams for proteinuria and serum creatinine. This policy enabled us to apply PP immediately after suspected FSGS recurrence and seemed to achieve a relatively higher remission rate of 87.5% than those of 50 to 70% in published studies [2, 5, 9]. Based on these results, we suggest that FSGS patients may benefit from careful monitoring, especially within two months after KT—because the median time of recurrence was within 1–2 months after transplantation [2, 12].

Our study is the first single-center study to report the preventable effects of prophylactic treatment against FSGS recurrence with statistical significance, which was possible due to the relatively large number of study patients, all of whom had histologically proven primary FSGS and had relatively homogeneously distributed study groups. Several studies have shown a tendency of a protective effect of pre-transplant treatment [4–6]. Ohta et al. reported that the prophylaxis group treated with 1 to 2 sessions of PP before KT appeared to have a lower recurrence rate (5 out of 15, 33.3%) than the non-prophylactic group (4 out of 6, 66.7%), although the small number of study patients limited the achievement of statistical significance [6]. Gohh et al. reported a recurrence rate of 30% with a course of 8 PP in the peri-operative period in high-risk patients with an expected recurrence rate of 60% [4]. Hickson et al. tried to find the evidence of prophylactic effects of preemptive PP followed by additional PP after KT; [5] although the study did not reach its goal, the authors observed that severe aspects of recurrent FSGS that do not respond to treatment such as primary non-function did not occur in patients who received preemptive treatment [5].

A major obstacle to studying FSGS is its low prevalence. Several studies using registry and meta-analysis have been performed to overcome the low sample size [2, 3, 18]. However, registry data had issues regarding data quality, missing data, and heterogeneity of transplant protocols among centers. During the study period (2007–2018), our center performed 4263 cases of KT, including 728 ABOi and 217 FXCM-positive KT, which allowed us to gather a sufficient number of FSGS cases and 28 pre-treatment recipients to have statistical strength. In addition, one physician at our center routinely performed prophylactic PP in FSGS, which allowed our cohort to have 23 patients in the pre-treatment group. The study groups in this study were divided homogeneously with the same clinical protocol, even though they were not randomly selected.

There were concerns that ABO incompatibility and FXCM-positivity could affect the clinical outcomes, and proteinuria with azotemia in the rejection episode was similar to the clinical aspect of recurred FSGS. However, we previously reported that ABOi KT had comparable graft survival and rejection rates after modifying the desensitization method, and recipients with pre-transplant DSA values less than 5000 MFI showed similar rejection rates with DSA-negative KT [13, 17]. Our multivariate analysis also revealed that ABO incompatibility was not a significant factor related to FSGS recurrence. Nevertheless, there is a possibility that rejection was confused with recurred FSGS because we did not perform graft biopsy in all cases due to the risk of bleeding complications, especially within 2 weeks after transplant. In addition, renal biopsies performed soon after FSGS recurrence mostly showed mild foot process effacement without definite segmental glomerular sclerosis [19]. We consider that this limitation would not have significantly affected the main conclusion of our study because a previous study in this cohort reported that the one-year rejection rate was less than 4% [17]. We performed a biopsy in cases that were challenging to distinguish between rejection and FSGS recurrence. As a result, one patient who had increased serum creatinine and proteinuria that did not fulfill the definition of recurred FSGS underwent graft biopsy at post-operative 13 days, which showed acute cellular and antibody-mediated rejection; the kidney function was restored after steroid pulse treatment and PP.

Patient selection is a critical factor in performing the study to determine the effect of pre-transplant treatment. The hypothesis is that a prophylactic treatment that removes a permeability factor decreases the risk of FSGS recurrence after KT and is utilized only when the study includes recipients with primary FSGS. We initially reviewed the records of every patient with FSGS and a native kidney biopsy report and discussed them with pathologists and nephrologists at our center. Patients with no definite foot process effacement in renal biopsy and absent nephrotic range proteinuria were excluded from our study. FSGS cases with possible secondary causes such as infection, hypertension, diabetes mellitus, and chronic renal failure due to other medical conditions were also excluded. Rituximab may have different mechanisms from PP in affecting the course of FSGS by modulating podocyte function [20]. Although one case report suggested protective effects of rituximab in preventing FSGS recurrence, there is still no conclusive study that demonstrated the efficacy of rituximab [21]. Our study also showed that rituximab had no significant effect as a prophylactic treatment.

The traditional risk factors for FSGS recurrence include native kidney failure within three years of onset, mesangial proliferation on biopsy, younger age, nephrectomy status, non-white ethnicity, and living donor transplantation [2, 4, 7]. Among them, only younger age was a significant factor in univariate analysis. Deceased donor transplantation was a significant factor in univariate analysis but not in multivariate analysis, which could be due to the fact that the living donor transplantation group included more pre-transplant treatment cases. Along with younger age, pre-transplant treatment remained statistically significant in multivariate analysis.

Younger age of FSGS onset is reported as a risk factor of recurrence in several studies [7, 8]. Likewise, younger age was a significant risk factor in our study. Given these findings, pediatric patients are more likely to be at higher risk of FSGS recurrence after transplantation as compared to adults. FSGS recurrence rate in pediatric patients was reported to be 14–60% in first transplants [19, 22]. It is difficult to directly compare these recurrence rates with the results of adult patients because each study had different inclusion criteria, races, immunosuppressive protocols, and definitions of disease recurrence. However, because there was no difference in the pathogenesis of FSGS between children and adults, our study may provide helpful information for pediatric patients. Pretransplant treatment has also been proposed in pediatric transplants [6, 23]. Ohta et al. suggested that prophylactic PP seemed to be effective in preventing FSGS recurrence, although the data did not achieve statistical significance due to a small number of case [6]. Verghese et al. introduced the center protocol that has applied pretransplant PP in pediatric FSGS patients since 2006 [23]. However, Verghese et al. did not find a significant reduction of disease recurrence following KT in the prophylactic PP group and hypothesized that PP was inadequate to reduce the circulation factor or related permeability factors related to FSGS recurrence [23].

Our study has several limitations. It has a retrospective design, and the treatment group was not selected in a randomized manner, thereby resulting in potential selection bias. Also, although we tried to exclusively include primary FSGS cases, there is a possibility that secondary FSGS cases were included in the analysis. Conversely, it is also possible that primary FSGS cases were excluded due to a lack of evidence. Second, we did not perform a protocol or indicational biopsy to evaluate the possible causes of azotemia and proteinuria in all cases with suspected FSGS recurrence. However, recent studies have enabled the diagnosis of recurrence and remission of FSGS without pathological confirmation when clinical aspects fulfill the criteria [15, 16]. Third, because desensitization in ABOi KT and FXCM-positive KT was not intended for prophylaxis for FSGS recurrence, the pre-transplant treatment included different protocols, including the number of PP and the use of rituximab and its dose. Fourth, we did not perform genetic analyses to find variants in susceptibility genes for development of FSGS, possibly resulting in different clinical manifestations and prognoses from primary FSGS. However, the clinical efficiency and cost-effectiveness of routine implementation of genetic studies in FSGS remain unclear [24].

## Conclusion

We found that pre-transplant treatment with PP had beneficial effects on preventing FSGS recurrence after KT. There is still a lack of guidelines on the prophylaxis for KT in FSGS. Although our study showed the beneficial effects of prophylactic treatment, a multinational randomized controlled study with a homogenous treatment protocol is warranted. We suggest that pre-transplant PP may be considered in FSGS cases undergoing KT, especially in those with a high risk for recurrence. In addition, KT recipients should be monitored carefully for 1–2 months after KT and immediately treated if they show any sign of FSGS recurrence.

## Data Availability

The datasets analyzed during the current study are available from the corresponding author on reasonable request.

## Abbreviations

FSGS: focal segmental glomerulosclerosis
KT: kidney transplantation
PP: plasmapheresis
ABOi: ABO-incompatible
FCXM: flow cytometry crossmatch
CR: complete remission
PR: partial remission
ATG: anti?thymocyte globulin
SD: standard deviation
IQR: interquartile range
OR: odds ratio
CI: confidence interval

## References

[1] Allen PJ, Chadban SJ, Craig JC, Lim WH, Allen RDM, Clayton PA, Teixeira-Pinto A, Wong G (2017). Recurrent glomerulonephritis after kidney transplantation: risk factors and allograft outcomes. Kidney Int.

[2] Uffing A, Pérez-Sáez MJ, Mazzali M, Manfro RC, Bauer AC, de Sottomaior Drumond F, O’Shaughnessy MM, Cheng XS, Chin KK, Ventura CG (2020). Recurrence of FSGS after kidney transplantation in adults. Clin J Am Soc Nephrol.

[3] Boonpheng B, Hansrivijit P, Thongprayoon C, Mao SA, Vaitla PK, Bathini T, Choudhury A, Kaewput W, Mao MA, Cheungpasitporn W (2021). Rituximab or plasmapheresis for prevention of recurrent focal segmental glomerulosclerosis after kidney transplantation: a systematic review and meta-analysis. World J Transplant.

[4] Gohh RY, Yango AF, Morrissey PE, Monaco AP, Gautam A, Sharma M, McCarthy ET, Savin VJ (2005). Preemptive plasmapheresis and recurrence of FSGS in high-risk renal transplant recipients. Am J Transplant.

[5] Hickson LJ, Gera M, Amer H, Iqbal CW, Moore TB, Milliner DS, Cosio FG, Larson TS, Stegall MD, Ishitani MB (2009). Kidney transplantation for primary focal segmental glomerulosclerosis: outcomes and response to therapy for recurrence. Transplantation.

[6] Ohta T, Kawaguchi H, Hattori M, Komatsu Y, Akioka Y, Nagata M, Shiraga H, Ito K, Takahashi K, Ishikawa N (2001). Effect of pre-and postoperative plasmapheresis on posttransplant recurrence of focal segmental glomerulosclerosis in children. Transplantation.

[7] Francis A, Trnka P, McTaggart SJ (2016). Long-term outcome of kidney transplantation in recipients with focal segmental glomerulosclerosis. Clin J Am Soc Nephrol.

[8] First MR (1995). Living-related donor transplants should be performed with caution in patients with focal segmental glomerulosclerosis. Pediatr Nephrol.

[9] Canaud G, Zuber J, Sberro R, Royale V, Anglicheau D, Snanoudj R, Gaha K, Thervet E, Lefrère F, Cavazzana-Calvo M (2009). Intensive and prolonged treatment of focal and segmental glomerulosclerosis recurrence in adult kidney transplant recipients: a pilot study. Am J Transplant.

[10] Savin VJ, Sharma R, Sharma M, McCarthy ET, Swan SK, Ellis E, Lovell H, Warady B, Gunwar S, Chonko AM (1996). Circulating factor associated with increased glomerular permeability to albumin in recurrent focal segmental glomerulosclerosis. N Engl J Med.

[11] Wada T, Nangaku M (2015). A circulating permeability factor in focal segmental glomerulosclerosis: the hunt continues. Clin Kidney J.

[12] Alasfar S, Matar D, Montgomery RA, Desai N, Lonze B, Vujjini V, Estrella MM, Manllo Dieck J, Khneizer G, Sever S (2018). Rituximab and therapeutic plasma exchange in recurrent focal segmental glomerulosclerosis Postkidney Transplantation. Transplantation.

[13] Kwon H, Kim YH, Choi JY, Sung S, Jung JH, Park SK, Han DJ (2016). Analysis of 4000 kidney transplantations in a single center: across immunological barriers. Med (Baltim).

[14] Pardon A, Audard V, Caillard S, Moulin B, Desvaux D, Bentaarit B, Remy P, Sahali D, Roudot-Thoraval F, Lang P (2006). Risk factors and outcome of focal and segmental glomerulosclerosis recurrence in adult renal transplant recipients. Nephrol Dial Transplant.

[15] Ban H, Miura K, Kaneko N, Shirai Y, Yabuuchi T, Ishizuka K, Chikamoto H, Akioka Y, Shimizu S, Ishida H (2021). Amount and selectivity of proteinuria may predict the treatment response in post-transplant recurrence of focal segmental glomerulosclerosis: a single-center retrospective study. Pediatr Nephrol.

[16] Troost JP, Trachtman H, Nachman PH, Kretzler M, Spino C, Komers R, Tuller S, Perumal K, Massengill SF, Kamil ES (2018). An Outcomes-Based definition of Proteinuria Remission in Focal Segmental Glomerulosclerosis. Clin J Am Soc Nephrol.

[17] Kwon H, Kim YH, Kim JY, Choi JY, Shin S, Jung JH, Park SK, Han DJ (2019). The results of HLA-incompatible kidney transplantation according to pre-transplant crossmatch tests: Donor-specific antibody as a prominent predictor of acute rejection. Clin Transpl.

[18] Briggs JD, Jones E (1999). Recurrence of glomerulonephritis following renal transplantation. Scientific Advisory Board of the ERA-EDTA Registry. European Renal Association-European Dialysis and Transplant Association. Nephrol Dial Transplant.

[19] Harshman LA, Bartosh S, Engen RM (2022). Focal segmental glomerulosclerosis: risk for recurrence and interventions to optimize outcomes following recurrence. Pediatr Transpl.

[20] Fornoni A, Sageshima J, Wei C, Merscher-Gomez S, Aguillon-Prada R, Jauregui AN, Li J, Mattiazzi A, Ciancio G, Chen L (2011). Rituximab targets podocytes in recurrent focal segmental glomerulosclerosis. Sci Transl Med.

[21] Audard V, Kamar N, Sahali D, Cardeau-Desangles I, Homs S, Remy P, Aouizerate J, Matignon M, Rostaing L, Lang P (2012). Rituximab therapy prevents focal and segmental glomerulosclerosis recurrence after a second renal transplantation. Transpl Int.

[22] Kim SJ, Ha J, Jung IM, Ahn MS, Kim M, Lee HS, Cheong HI, Choi Y (2001). Recurrent focal segmental glomerulosclerosis following renal transplantation in korean pediatric patients. Pediatr Transpl.

[23] Verghese PS, Rheault MN, Jackson S, Matas AJ, Chinnakotla S, Chavers B (2018). The effect of peri-transplant plasmapheresis in the prevention of recurrent FSGS. Pediatr Transpl.

[24] Rosenberg AZ, Kopp JB (2017). Focal segmental glomerulosclerosis. Clin J Am Soc Nephrol.

